# Optimization of Illuminance and Color-Temperature Conditions for Railway Passengers’ Comfort Based on Oxygenated Hemoglobin Saturation in the Brain

**DOI:** 10.3390/toxics13030212

**Published:** 2025-03-14

**Authors:** Minkyeong Kim, Jooyeon Lee, Yonghee Lee, Duckshin Park

**Affiliations:** 1 Railroad Test & Certification Division, Korea Railroad Research Institute (KRRI), Cheoldo Bangmulgwan-ro, Uiwang-si 16105, Republic of Korea; mkkim15@krri.re.kr; 2 Transportation Environmental Research Department, Korea Railroad Research Institute (KRRI), Cheoldo Bangmulgwan-ro, Uiwang-si 16105, Republic of Korea; jooyeon07@krri.re.kr; 3 Global Institute for Advanced Nanoscience & Technology (GIANT), Changwon National University, Changwon-si 51140, Republic of Korea; leedragon83@gmail.com

**Keywords:** brain activation, comfort, fNIRS, light and color temperature, GLM

## Abstract

Railway travel is an eco-friendly means of transportation, and passengers are spending increasing amounts of time on trains while engaging in various activities. As a major factor affecting railway passengers’ comfort, we investigated the effects of lighting. Korean train cars are required to have two rows of light-emitting diode lights with a minimum illuminance of 500 lx, so we examined changes in cerebral blood flow under various illuminance conditions around this threshold value. We used functional near-infrared spectroscopy to measure prefrontal cortex activation in 29 college students under illuminance values of 300, 500, and 800 lx and color-temperature values of 2700 K (bulb color), 4000 K (white color), and 5500 K (blue color). Mean brain activity values were compared using analysis of variance. Of the 48 channels, significant interaction effects between color and illuminance on brain activation responses were observed for channel 38, as well as in the right dorsolateral prefrontal cortex among the different brain regions of the Brodmann area (*p* < 0.05). Oxygenated hemoglobin concentrations had consistently negative values for all the treatment combinations, and individual treatment analyses based on single-sample Student’s *t*-tests showed different degrees of brain activation among channels and Brodmann areas. Meanwhile, a comparison of absolute values indicated that an illuminance level of 500 lx was more comfortable than levels of 300 and 800 lx, and that white color was more comfortable than bulb color and blue color. These results provide a scientific basis for the design of train cars that improve passenger comfort and satisfaction, which is anticipated to enhance the quality of railway services.

## 1. Introduction

The use of public transportation is continuously increasing, with high-speed rail and urban rail travel gaining attention as efficient and eco-friendly transportation modes. In 2024, 116.58 million people used high-speed railways in Korea; in particular, the Seoul Urban Railway is a major means of public transportation, with 8 million daily users [1]. Seoul citizens are reported to spend 33 min/day on public transportation [2]. The role of passengers’ comfort in maintaining their health and stability through various activities, such as reading, smartphone use, sleeping, and resting in indoor spaces, implies that rail travel can be more than simply a means of transportation. As passengers spend more time in railway cars, the lighting environment is receiving attention as a factor that can improve passengers’ comfort.

Environmental factors within a train car, particularly illuminance and its color temperature, can significantly impact the psychological and physiological responses of passengers. Previous studies have demonstrated that appropriate lighting can reduce fatigue and improve concentration, and that color temperature can affect mood and cognitive functions [3,4,5,6]; these factors contribute to overall passenger satisfaction. However, there is a lack of experimental data on the effects of these environmental factors on the comfort of railway passengers. Previous studies [3,4,5,6] have examined various effects of lighting conditions on the human body [7,8,9,10], but systematic investigations of specific methods for improving comfort within a train car are lacking. In a rapidly changing public transportation environment, collecting and analyzing physiological data are essential for interpreting passengers’ comfort.

In Korea, train cars are required by the Korean standard for illumination in railway vehicles (KS R no. 9159) to have two rows of light-emitting diode (LED) lights, with a minimum illuminance of 500 lx. This criterion was not presented based on specific experiments. Qualitative methods such as questionnaire surveys have limitations that include subjective evaluation, imprecision, and dependence on memory. Measurement of changes in cerebral blood flow would be an appropriate method for determining the relationship between illumination and passenger comfort. Functional near-infrared spectroscopy (fNIRS) provides noninvasive measurements of blood oxygenation in the cerebral cortex, changes in which can reveal the effects of illuminance and color temperature on specific areas of the brain [11,12,13,14,15,16,17,18].

The prefrontal cortex, the subject of our study using fNIRS, is a large region located at the front of the brain that processes cognitive functions and emotional responses. Blood flow in the prefrontal lobe tends to decrease in response to positive emotions and increase in response to negative emotions and stress [19,20,21,22]. The use of prefrontal lobe activity as a reliable indicator in many studies implies that it is highly suitable for analyzing brain responses to illuminance and color temperature within railway vehicles.

Therefore, we investigated the optimal lighting conditions for railway passengers’ comfort by using fNIRS technology to monitor changes in brain activity in real time, with the aim of promoting health and stability during rail travel. The results of this study provide basic scientific data for the design of comfortable train cars, as well as practical information for improving passenger satisfaction and enhancing the quality of railway services and user-centered interior design. Our findings will play an important role in strengthening the overall competitiveness of the public transportation system.

## 2. Materials and Methods

### 2.1. Experimental Conditions

**Figure 1:**
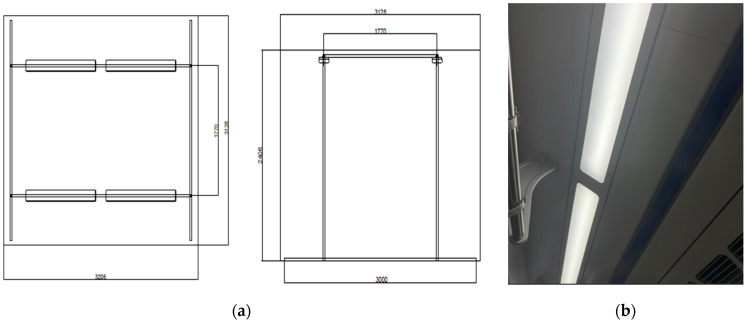
Lighting system configuration within the experimental chamber (**a**) and real train ceiling lighting (**b**).

**Figure 2:**
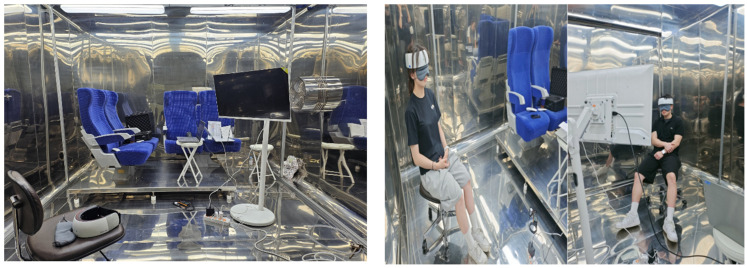
Experimental chamber setup.

The experimental setting consisted of a large chamber (area, 24 m^3^; 3.206 m × 3.128 m × 2.406 m) in which environmental variables could be controlled. A separate waiting area was maintained outside the chamber to avoid interfering with the brain-wave experiment whenever possible. The chamber was designed to replicate the environment of a train car, with ceiling lighting comprising four 75 W LED light sources configured in two rows (Figure 1). The lights could be adjusted to 2700 K (bulb color), 4000 K (white color), or 5500 K (blue color). The illuminance reaching a given subject’s seat was set to 300, 500, or 800 lx. The temperature within the chamber was maintained at 26 °C, and the relative humidity at 60%. A 43″ monitor was installed at eye level (Figure 2). For illuminance measurements, we used a calibrated lux meter with a measurement range of 0–100,000 lx (Testo 545; Testo, Titisee-Neustadt, Germany).

### 2.2. Experimental Design

**Figure 3:**
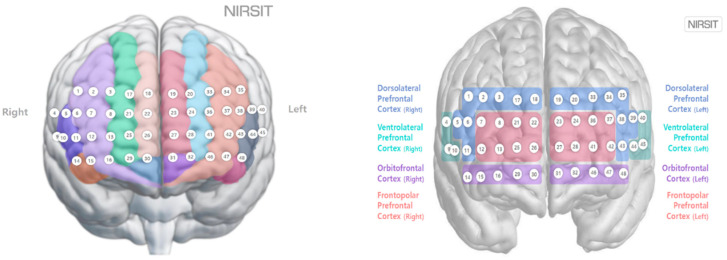
The 48 channels and Brodmann mapping of the NIRSIT channels.

The experiment was conducted over three days between 13 and 16 August 2024, with 10 subjects tested per day. The experiment consisted of combinations of three illuminance conditions (300, 500, and 800 lx) and three color-temperature conditions (2700, 4000, and 5500 K). Each subject provided informed consent after hearing an explanation of the experimental procedure. A flowchart of the experiments is shown in Figure 3. Each subject rested in the waiting room for 10 min prior to the experiment and then entered the chamber and sat on a chair facing the monitor. Then, the researcher prepared the environment for the experimental conditions to be tested. For prefrontal cortex measurements, a NIRSIT Lite wireless fNIRS device (OBELAB, Seoul, Republic of Korea) operating with 48 channels was attached to the center of the subject’s forehead, 1 cm above the eyebrows, such that no light could enter the device; the subject’s hair was pushed back as much as possible. The 48 channels corresponded to the left and right brains of the frontal lobe as shown in Figure 3, and the Brodmann area was divided into the left and right dorsolateral prefrontal cortex, ventrolateral prefrontal cortex, orbitofrontal cortex, and frontopolar prefrontal cortex (OBELAB) areas.

**Figure 4:**
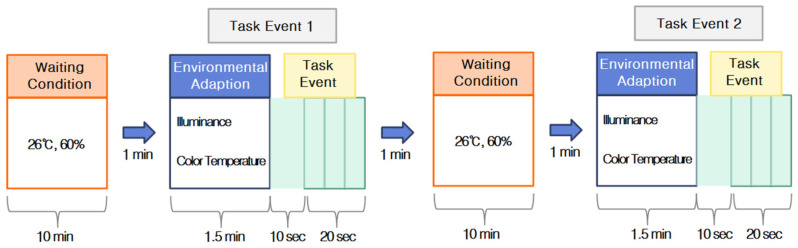
Flowchart of the experiments.

Calibration was performed for each subject during the experiment, and the subject was asked to maintain comfortable body and head postures and to minimize head movement. Data were recorded using the NIRSIT SCAN v1.3 software (OBELAB), beginning at the start of the experiment. The subject wore a blindfold for 1 min to stabilize brain activity, after which the blindfold was removed, light stimulation was provided for 1.5 min, and a task was performed for 30 s. The task was designed to be easy and to require approximately 30 s based on preliminary experiments. The task showed guide maps frequently seen in railway stations and subways and presented questions related to the information on the guide maps. Each subject repeated the same procedure for each combination of illuminance and color-temperature treatments for a total of approximately 30 min (Figure 4). After each experiment, the subject was taken to a separate area and asked to fill out a survey that included questions about their basic demographic information (e.g., name, sex, and age) and the subject’s emotional responses to the different lighting and color-temperature conditions.

### 2.3. Features of the fNIRS Test Device 

Through fNIRS, it is possible to determine which parts and areas of the cerebral cortex should be considered in terms of environmental variables such as illuminance and color temperature, and which environmental conditions lead to comfort, value judgment, and other types of brain activity. In contrast, magnetic resonance imaging studies can provide high-resolution images, but they are expensive and time-consuming. Compared with electroencephalography (EEG) [3,6,7,8,23,24,25,26,27,28], which is a relatively inexpensive and portable technology that provides ionophysiological measurements of brain surface activity, fNIRS can monitor hemodynamic changes in real time by irradiating the cerebral cortex with two wavelengths of NIR light (780 and 850 nm) [11,12,13,14,15,16,17]. NIR spectral light is unique in its ability to pass through tissues and its preferential absorption by hemoglobin in the cerebral cortex [18,29]. The absorbance spectrum of hemoglobin varies depending on its binding to oxygen, which allows the fNIRS device to continuously detect relative changes in oxygenated hemoglobin (oxyhemoglobin) and deoxygenated hemoglobin (deoxyhemoglobin) in cortical areas [18,30]. Due to neurovascular coupling, the fNIRS signal can be considered a surrogate measure of basal neural activity [18,31]. Local neural activity induces blood flow and volume increases that are several times higher than metabolic demand. Therefore, cerebral hemodynamic responses are generally associated with large increases in oxyhemoglobin; concomitantly, there is an increase in deoxyhemoglobin and a slight decrease in hemoglobin [18,32]. The resulting net changes in oxyhemoglobin make it suitable as a marker of brain activity [18,33]. Although NIR light cannot reach subcortical brain regions, this noninvasive procedure can map brain activity by measuring changes in hemoglobin concentrations [18,34].

### 2.4. Participants

A total of 29 college students (15 men, 14 women; average age, 22.8 ± 3.3 years) participated in the experiments. All subjects were physically healthy with no history of disease; any subject who was unwell, such as having COVID-19, on the day of the pre-survey and examination was excluded from the study. Subjects with color-perception problems such as color blindness or color weakness, who had undergone laser eye surgery, with a history of neurological disease or sensory abnormalities, or exhibiting difficulty in physical activities or communication were also excluded. The number of subjects required to achieve the research objective was calculated using the G*power program 3.1.9.7. Based on the calculation results for a significance level of 0.05, a power of 0.95, and 3 groups, a minimum of 24 research participants was required [35,36]. The subjects wore short-sleeved shirts and shorts, as the study was conducted during summer. The study protocol was reviewed by a joint institutional bioethics committee in accordance with the guidelines of the Declaration of Helsinki (P01-202408-01-015).

### 2.5. Signal Preprocessing

Data preprocessing was performed using the NIRSIT Quest v1.1.2 software (OBELAB). On the basis of *t* values, the fNIRS method reveals quantitative trends in the degree of frontal lobe activity in response to exposure to illuminance and color temperature from images. Signal preprocessing is performed in the following order on results obtained from the NIRSIT SCAN data acquisition tool: handling invalid values, channel rejection, data conversion, motion artifact removal, data conversion, digital filtering, channel rejection, and rejection padding. Handling invalid values in raw intensity data is performed by applying nearest-neighbor interpolation if there are no more than five subsequent invalid cases; otherwise, channels are rejected. Channel rejection is performed on raw intensity data by rejecting those channels with a median intensity < 30 or a coefficient of variation > 25% and identical consecutive values comprising >5% of the entire time series (indicating saturation). This fNIRS device is a system with measurement noise of about 1 to 5 Arbitrary Units (A.U.), and in the case of the 30 Arbitrary Units (A.U.) set in this study, it is a very minimum threshold with an SNR of about 15 dB. First, this criterion was used as the first cutoff to filter out unreliable signals, and then, with additional criteria such as coefficient of variation (CV), we tried to apply an empirical criterion that fits the data characteristics.

Data conversion of raw intensity data is performed by converting light intensity into optical density. Motion artifact removal is performed on hemoglobin concentration data through temporal derivative distribution repair. Optical density data are converted to hemoglobin concentration data (mm × mM) based on ISO guidelines; concentration changes are calculated using molar extinction coefficients following the method of Moaaveni [11,12], without accounting for differential path lengths. Digital filtering of the hemoglobin concentration data is applied to remove frequencies of no interest (i.e., potential physiological noise or measurement noise/drift), based on a discrete cosine transform bandpass filter, with lower and upper cutoff frequencies of 0.005 and 0.1 Hz, respectively. The second round of channel rejection is performed on hemoglobin concentration data by rejecting channels with oxyhemoglobin and deoxyhemoglobin levels demonstrating extreme negative correlations (mirroring) (i.e., R < −0.9). Finally, rejection padding is performed by replacing missing channels with values from backup channels, which may be mean values from Brodmann-area channels. In the present study, we examined extinction coefficients and applied an average channel rejection ratio less than 5%.

### 2.6. Statistical Analyses

Generally, fNIRS data are analyzed using block averaging, general linear model (GLM) analysis, and/or connectivity analysis. In this study, we applied a GLM, which is a holistic approach that considers multiple influences on the entire dataset and that allows estimates to be obtained while controlling for the effects of major factors. Our GLM analysis was conducted at both the individual subject and group levels.

At the subject level, signal normalization was not selected to examine group effects, and an autoregressive iterative reweighted least-squares algorithm was applied for the autocorrelation removal to consider extreme data values, using the hrf + temporal derivative + dispersion derivative settings. Finally, angles x, y, and z were entered into the nuisance regressor, and a short separation channel (≤15 mm) was determined based on the channel mean. For the group-level analysis, we applied two-way analysis of variance (ANOVA) using the NIRSIT Quest v1.1.2 software; the results were expressed as brain activation maps. This study had three conditions for illuminance and color temperature, so after ANOVA analysis, a one-sample *t*-test was performed to analyze the degree of brain activation for each condition. The one-sample *t*-test is a test to determine whether the mean of the sample is different from a specific value. The null hypothesis is that there is no significant difference in the conditions, and the alternative hypothesis is that there is a significant difference in the conditions. The aim was to compare the average value of each group.

## 3. Results

### 3.1. Comparison of Illuminance and Color-Temperature Treatment Responses

In this study, we considered two variables, illuminance and color temperature, and compared the data from three groups to verify their effects on each other. For the channels, we obtained mean oxyhemoglobin concentrations of −0.005 ± 0.062 mm × mM for an illuminance of 300 lx, −0.004 ± 0.065 mm × mM for 500 lx, and −0.006 ± 0.064 mm × mM for 800 lx; for the Brodmann area, we obtained mean oxyhemoglobin concentrations of −0.004 ± 0.040 mm × mM for an illuminance of 300 lx, −0.003 ± 0.042 mm × mM for 500 lx, and −0.004 ± 0.041 mm × mM for 800 lx. For the channels, we obtained mean oxyhemoglobin concentrations of −0.007 ± 0.063 mm × mM for bulb color, −0.002 ± 0.065 mm × mM for white color, and −0.006 ± 0.063 mm × mM for blue color; for the Brodmann area, we obtained oxyhemoglobin concentrations of −0.006 ± 0.040 mm × mM for bulb color, −0.002 ± 0.041 mm × mM for white color, and −0.003 ± 0.043 mm × mM for blue color. The results represent the mean oxyhemoglobin concentration for all 48 channels, with no channels being rejected. Thus, all mean illuminance and color-temperature values were negative, with differences in the degree of activation demonstrated in the cerebral hemodynamic response.

**Figure 5:**
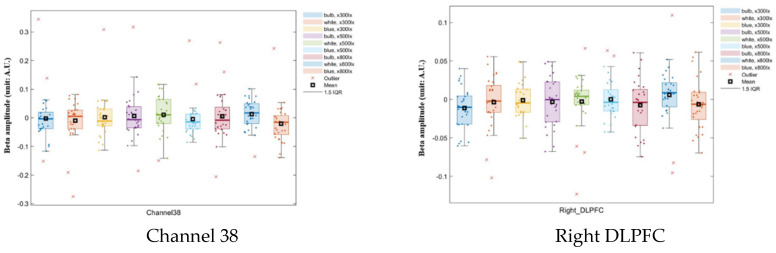
Mean oxyhemoglobin concentrations in channel 38 and the dorsolateral prefrontal cortex (DLPFC) using two-way ANOVA analysis.

ANOVA results showed that channel 38 values were significantly different from the values of all other channels for all combinations of illuminance and color-temperature conditions (*p* < 0.05); there is a significant interaction effect between color and illumination on the oxyhemoglobin concentration in channel 38. And the Brodmann area, the right DLPFC produced significantly different results from all the other regions (*p* < 0.05; Table 1 and Table 2; Figure 5). The y-axis represents the parameter estimate, i.e., beta amplitude, and the unit is Arbitrary Units (A.U.). Outliers were identified using the IQR (interquartile range). This frontal lobe region is involved in work processes and reasoning ability.

**Figure 6:**
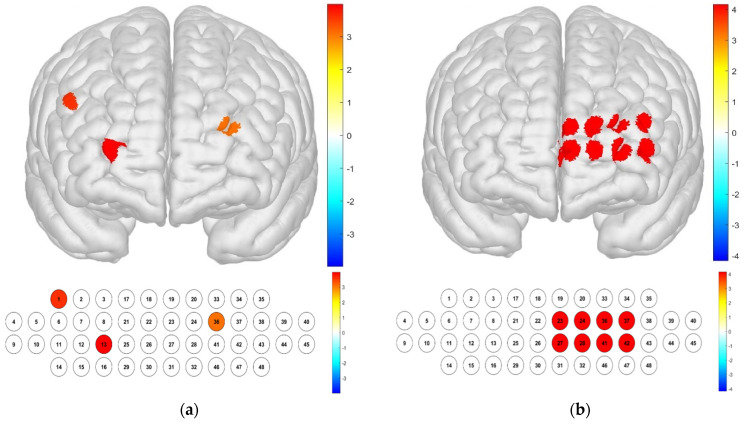
Brain activation analysis of responses to illuminance treatments for different channels (**a**) and the Brodmann area (**b**). (Two-way ANOVA on HbO concentration for illuminance; *p*-value from Student’s *t*-test (uncorrected *p* < 0.05)).

Significant differences among illumination treatments were confirmed in channels 1, 13, and 36. For the Brodmann area, significant differences were confirmed in the left FPC (Figure 6), which is a frontal lobe region that plays a role in metacognition and decision-making processes such as observation, control, and judgment, which may be influenced by illumination conditions.

**Figure 7:**
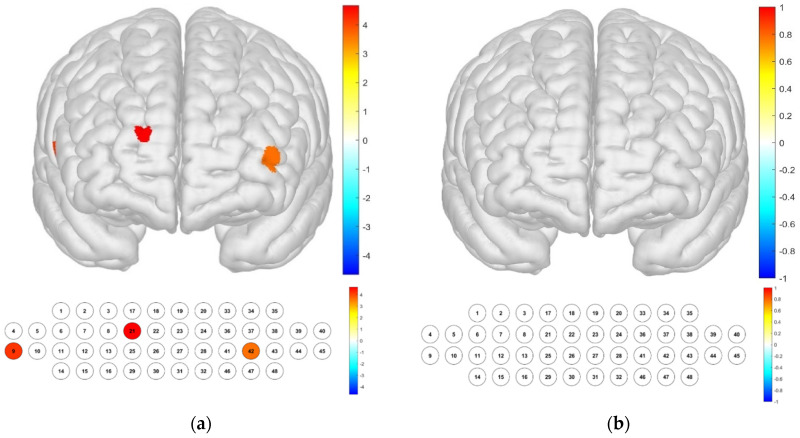
Brain activation analysis of responses to color-temperature changes for different channels (**a**) and the Brodmann area (**b**). (Two-way ANOVA on HbO concentration for color-temperature; *p*-value from Student’s *t*-test (uncorrected *p* < 0.05)).

In terms of differences according to color temperature, it was confirmed that significant differences appeared in channels 9, 21, and 42, which were not the same as for illuminance. When examining the Brodmann area, it was confirmed that there was no area in which significant differences appeared (Figure 7).

### 3.2. Cerebral Blood-Flow Responses to Different Illumination and Color-Temperature Conditions

Because the degree of brain activation for each condition could not be examined through ANOVA analysis alone, we analyzed the data for the nine conditions individually using single-sample Student’s *t*-tests.

**Figure 8:**
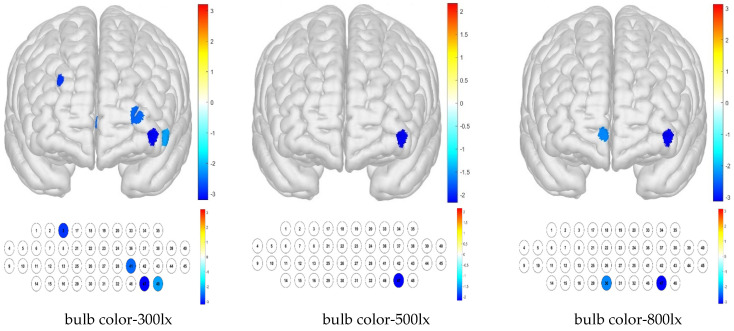
Brain activation analysis of responses to bulb color and illuminance treatments for different channels (one-sample Student’s *t*-test results for HbO concentration; *p*-value from Student’s *t*-test (uncorrected *p* < 0.05)).

The one-sample *t*-test results show that when the sample mean is lower than other channels by condition, it can be deduced that the activation is small. Thus, low average activation was obtained for the bulb color–300 lx combination because of the contributions of channels 3, 41, 46, and 48; for the bulb color–500 lx combination, this was due to that of channel 47; and for the bulb color–800 lx combination, these were due to those of channels 30 and 46 (Figure 8).

**Figure 9:**
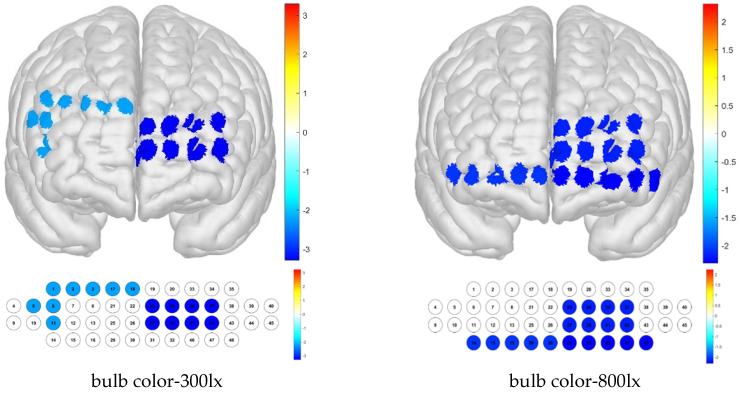
Brain activation analysis of responses to bulb color and different illuminance treatments for the Brodmann area (one-sample Student’s *t*-test results for HBo concentration; *p*-value from Student’s *t*-test (uncorrected *p* < 0.05)).

For the Brodmann area, our results demonstrate that the right DLPFC and left FPC regions had lower levels of activation under the combination of bulb color temperature and an illuminance of 300 lx. In contrast, the brain activity in response to the combination of bulb color temperature and 500 lx illuminance was not significantly different from the sample average. Little brain activation was detected in the OFC or left FPC regions for the combination of bulb color temperature and 800 lx illuminance (Figure 9).

**Figure 10:**
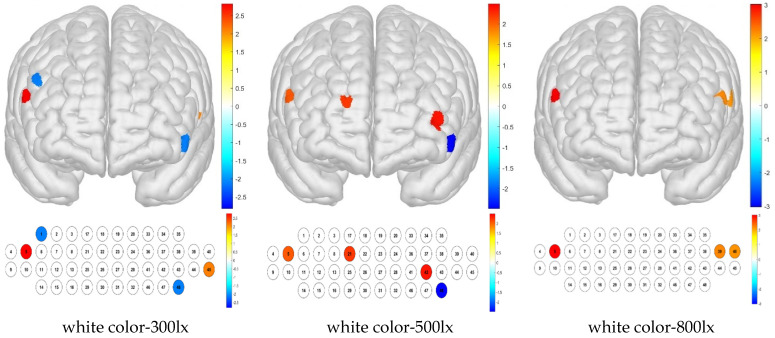
Brain activation analysis for white temperature color and different illuminance levels for different channels (one-sample Student’s *t*-test results for HBo concentration; *p*-value from Student’s *t*-test (uncorrected *p* < 0.05)).

The combination of white color temperature and 300 lx illuminance resulted in low brain activity sample averages for channels 1 and 48, whereas activation was high in channels 5 and 45. The combination of white color temperature and 500 lx illuminance produced a low sample average in channel 48 but high activation in channels 5, 21, and 42. The white–800 lx combination resulted in high brain activation in channels 5, 39, and 40 (Figure 10).

For the Brodmann area, no significant differences from the sample average were detected for white color temperature in combination with any illuminance level.

**Figure 11:**
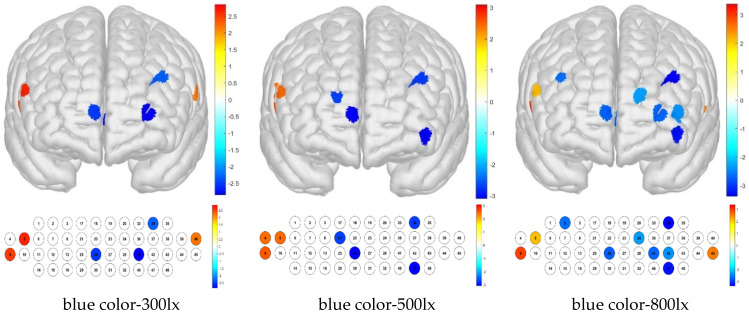
Brain activation analysis of responses to blue light and different illuminance levels for different channels (one-sample Student’s *t*-test results for HBo concentration; *p*-value from Student’s *t*-test (uncorrected *p* < 0.05)).

For treatment combinations including blue light, 300 lx illuminance produced low brain activation responses for channels 26, 34, and 41 and high brain activation responses for channels 5, 9, and 40; 500 lx illuminance produced low brain activation responses for channels 21, 26, 34, and 47 and high brain activation responses for channels 4, 5, and 9; and 800 lx illuminance produced low brain activation responses for channels 2, 24, 26, 34, 41, 42, and 47 and high brain activation responses for channels 5, 9, and 45 (Figure 11).

**Figure 12:**
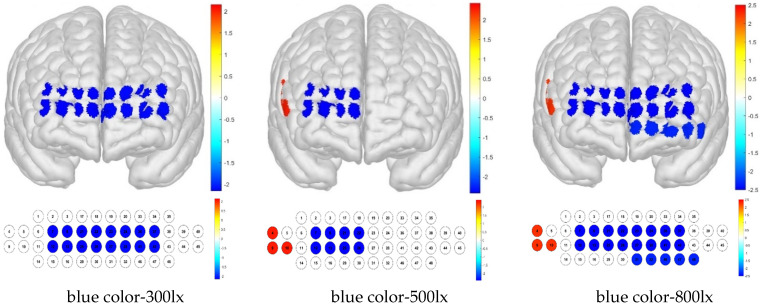
Brain activation analysis of responses to blue light and different illuminance levels in the Brodmann area (one-sample Student’s *t*-test results for HBo concentration; *p*-value from Student’s *t*-test (uncorrected *p* < 0.05)).

For the Brodmann area, responses to blue light under various illuminance levels were mainly low, whereas those to simulated daylight were high in some regions at illuminance levels of 500 and 800 lx. For the blue light–300 lx combination, brain activity was low in the FPC, which is related to metacognition and decision-making. For the blue light–500 lx illuminance combination, activity was low in the right FPC and high in the right VLPFC. For the blue light–800 lx combination, brain activity was low in the left OFC and FPC, and high in the right VLPFC (Figure 12).

## 4. Discussion

The experimental setup was designed to emulate the passenger compartment of a train, with a lighting scheme based on Korean regulations for railway lighting. Thus, LED lights were installed in two rows, and illuminance levels around the threshold of 500 lx were tested. Based on the experimental data, we analyzed the effects of combined illuminance and color-temperature treatments on railway passengers’ comfort based on changes in blood flow in the cerebral cortex, with the aim of determining optimal lighting conditions for comfortable railway travel. Whereas previous studies were based on subjective evaluations of questionnaire responses, this study integrated both fNIRS and questionnaire data to analyze physiological responses. Our results revealed consistently negative oxyhemoglobin concentrations under all combinations of illuminance and color temperature, while a comparison of absolute values indicated that an illuminance level of 500 lx was more comfortable than levels of 300 and 800 lx, and that white color was more comfortable than bulb color and blue color.

Previous studies related to illuminance and color temperature have focused on the indoor lighting environment of buildings [3,5,7,27,37] and the psychological [6,25,28,38] and physiological [8,26,28,38] effects of color temperature and illuminance. Some studies have investigated the illuminance and color temperature of interior lighting in automobiles, and others have examined psychophysiological responses to illuminance and color temperature using EEG, electrocardiography (ECG), and galvanic skin responses during resting, reading, and viewing activities in vehicles [22], including one study that identified the color temperatures that provided comfort to users of autonomous vehicles, based on EEG and pulse photoplethysmography [10]. However, no study related to passenger comfort in train cars has examined the effects of LED lighting or the optimal lighting for passenger activities, or obtained quantitative data based on fNIRS.

The prefrontal cortex is divided into four regions: the dorsolateral prefrontal cortex (DLPFC), ventrolateral prefrontal cortex (VLPFC), frontopolar prefrontal cortex (FPC), and orbitofrontal cortex (OFC). The DLPFC is responsible for working memory and reasoning ability; the VLPFC is responsible for working memory, language ability, and attention; the FPC is related to metacognition and decision-making; and the OFC is involved in emotional regulation and reward processing [39,40].

For the Brodmann area, our results demonstrate that the right DLPFC and left FPC regions had lower levels of activation under a combination of bulb color temperature and an illuminance of 300 lx. The DLPFC affects working memory and reasoning ability, whereas the FPC affects metacognition and decision-making. Little brain activation was detected in the OFC or left FPC regions for the combination of bulb color temperature and 800 lx illuminance. The OFC is related to emotional regulation and reward processing, and the FPC affects metacognition and decision-making. For the blue light–500 lx illuminance combination, activity was low in the right FPC and high in the right VLPFC; the latter region is related to working memory, language ability, and attention. For the blue light–800 lx combination, brain activity was low in the left OFC and FPC and high in the right VLPFC. The OFC is related to emotional regulation and reward processing.

In the early stages, many studies on lighting were conducted based on EEGs, and studies on lighting were mainly conducted on the interior of buildings. In addition, although some vehicles were examined for appropriate illumination and color-temperature conditions, no studies were conducted on the optimal conditions for illumination and color temperature inside railway vehicles, which are covered in this study. In actual research cases, illuminance and color-temperature conditions were set for each criterion, and in this study, there was a condition of 500 lx for the railway vehicle criterion, so the values before and after were set based on this. In other studies, illuminance varied from 3 to 1000 lx, and color temperature was set to only one value, or in some cases, it was presented as bulb color, white color, and blue color, which are the same as this study. However, the target-setting conditions are different. This study was not a qualitative study based on a questionnaire but a quantitative study based on the degree of activation of the frontal lobe. In addition, this study is different from other studies in that it performed ANOVA analysis by setting three conditions for illumination and color temperature and performed one-sample *t*-tests for each condition to derive the results. Based on the results showing high or low activity in specific channels and Brodmann areas, conditions for feeling relative stress and comfort under certain conditions were derived. Based on these results, the optimal conditions felt by passengers in railway vehicles can be reflected in the vehicle design.

The application of fNIRS technology requires static calibration of the equipment used to measure blood flow in the cerebral cortex, and correct fixation of the equipment to the subject is important. To ensure good signal data, the subject must refrain from head movements as much as possible when a question is asked and when putting on and taking off the blindfold.

## 5. Conclusions

The physiological response patterns indicated that the prefrontal cortex was significantly affected under various combinations of illuminance and color temperature in channel 38 and in the right DLPFC of the Brodmann area, which affects reasoning and work. Examining the individual effects of color temperature and illuminance revealed significant effects on the prefrontal cortex in various channels and on regions of the Brodmann area. Notably, illuminance changes had significant effects in the left FPC, confirming that differences in illuminance affect decision-making. This study is the first to apply fNIRS techniques to a railway environment and to clarify which channels and Brodmann areas may be affected by illuminance and color temperature. The degree of brain activation in highly activated channels and in the Brodmann area differed under nine treatment combinations: incandescent color, neutral white, and simulated daylight under three different illuminance settings. Therefore, we infer that passengers’ comfort can be improved by considering the functions of each brain region and channel influenced by these treatment conditions.

The findings of this study demonstrate that color-temperature and illuminance conditions affect railway passengers, that it is possible to analyze such effects on the frontal lobe of the brain statistically, and that the analysis can be conducted through a complementary approach that includes experimental measurements and survey questionnaires.

In this study, there was a limitation in signal processing due to the limitation of some heads moving when wearing the blindfold without removing it. In addition, although the majority of the participants in this study were aged in their 20s, different results may be derived depending on the age group. In addition, since the conditions that passengers feel comfortable in may be the conditions that they are used to, additional consideration of this factor will also be required. Future studies of the effects of lighting illuminance and color temperature on railway passengers’ comfort may provide further insights by expanding the range of environmental variables examined, such as heat, odors, and wind speed, and applying weights to various conditions based on comprehensive experiments.

## Figures and Tables

**Table 1:** Analysis of variance (ANOVA) results for each channel.

Index	Effect	df_1_	df_2_	F	*p*
Channel 1	Color	2	168	0.103	0.903
	Illumination	2	168	3.603	0.031 *
	Color × Illumination	4	168	0.450	0.767
Channel 2	Color	2	168	0.348	0.707
	Illumination	2	168	0.264	0.766
	Color × Illumination	4	168	2.241	0.068
Channel 3	Color	2	168	0.732	0.484
	Illumination	2	168	0.317	0.689
	Color × Illumination	4	168	0.707	0.564
Channel 4	Color	2	168	1.588	0.210
	Illumination	2	168	0.899	0.408
	Color × Illumination	4	168	1.850	0.122
Channel 5	Color	2	168	2.890	0.061
	Illumination	2	168	0.229	0.791
	Color × Illumination	4	168	0.074	0.989
Channel 6	Color	2	168	0.589	0.557
	Illumination	2	168	0.974	0.372
	Color × Illumination	4	168	1.008	0.400
Channel 7	Color	2	168	0.168	0.845
	Illumination	2	168	1.146	0.318
	Color × Illumination	4	168	0.407	0.791
Channel 8	Color	2	168	0.619	0.541
	Illumination	2	168	1.074	0.344
	Color × Illumination	4	168	0.562	0.689
Channel 9	Color	2	168	4.185	0.019 *
	Illumination	2	168	0.117	0.881
	Color × Illumination	4	168	0.553	0.690
Channel 10	Color	2	168	0.176	0.839
	Illumination	2	168	0.504	0.596
	Color × Illumination	4	168	0.876	0.475
Channel 11	Color	2	168	0.038	0.963
	Illumination	2	168	2.323	0.102
	Color × Illumination	4	168	0.542	0.703
Channel 12	Color	2	168	0.055	0.947
	Illumination	2	168	0.126	0.878
	Color × Illumination	4	168	1.352	0.253
Channel 13	Color	2	168	0.581	0.562
	Illumination	2	168	3.989	0.021 *
	Color × Illumination	4	168	0.447	0.774
Channel 14	Color	2	168	1.337	0.268
	Illumination	2	168	0.604	0.537
	Color × Illumination	4	168	1.279	0.282
Channel 15	Color	2	168	0.123	0.885
	Illumination	2	168	1.174	0.310
	Color × Illumination	4	168	1.286	0.278
Channel 16	Color	2	168	0.039	0.962
	Illumination	2	168	1.331	0.267
	Color × Illumination	4	168	0.942	0.439
Channel 17	Color	2	168	0.159	0.853
	Illumination	2	168	0.650	0.521
	Color × Illumination	4	168	0.080	0.988
Channel 18	Color	2	168	0.256	0.775
	Illumination	2	168	0.587	0.491
	Color × Illumination	4	168	1.248	0.295
Channel 19	Color	2	168	0.276	0.759
	Illumination	2	168	0.226	0.784
	Color × Illumination	4	168	0.958	0.428
Channel 20	Color	2	168	0.302	0.740
	Illumination	2	168	1.027	0.359
	Color × Illumination	4	168	0.261	0.899
Channel 21	Color	2	168	4.665	0.012 *
	Illumination	2	168	0.039	0.927
	Color × Illumination	4	168	0.933	0.428
Channel 22	Color	2	168	1.571	0.214
	Illumination	2	168	1.156	0.310
	Color × Illumination	4	168	0.757	0.531
Channel 23	Color	2	168	0.201	0.818
	Illumination	2	168	0.100	0.866
	Color × Illumination	4	168	1.199	0.314
Channel 24	Color	2	168	0.167	0.846
	Illumination	2	168	1.413	0.246
	Color × Illumination	4	168	0.604	0.631
Channel 25	Color	2	168	0.382	0.684
	Illumination	2	168	0.774	0.449
	Color × Illumination	4	168	0.423	0.770
Channel 26	Color	2	168	3.046	0.053
	Illumination	2	168	1.526	0.222
	Color × Illumination	4	168	1.841	0.130
Channel 27	Color	2	168	0.761	0.470
	Illumination	2	168	0.621	0.497
	Color × Illumination	4	168	2.350	0.075
Channel 28	Color	2	168	0.458	0.634
	Illumination	2	168	0.504	0.597
	Color × Illumination	4	168	0.186	0.940
Channel 29	Color	2	168	0.407	0.667
	Illumination	2	168	1.284	0.280
	Color × Illumination	4	168	1.243	0.295
Channel 30	Color	2	168	0.612	0.544
	Illumination	2	168	0.418	0.647
	Color × Illumination	4	168	1.049	0.382
Channel 31	Color	2	168	0.372	0.690
	Illumination	2	168	1.311	0.272
	Color × Illumination	4	168	0.242	0.908
Channel 32	Color	2	168	1.694	0.190
	Illumination	2	168	0.316	0.728
	Color × Illumination	4	168	0.547	0.700
Channel 33	Color	2	168	0.284	0.754
	Illumination	2	168	2.534	0.083
	Color × Illumination	4	168	0.292	0.881
Channel 34	Color	2	168	1.284	0.282
	Illumination	2	168	2.613	0.081
	Color × Illumination	4	168	0.580	0.665
Channel 35	Color	2	168	0.459	0.634
	Illumination	2	168	1.132	0.318
	Color × Illumination	4	168	0.740	0.543
Channel 36	Color	2	168	0.461	0.632
	Illumination	2	168	3.119	0.047 *
	Color × Illumination	4	168	1.560	0.187
Channel 37	Color	2	168	0.078	0.925
	Illumination	2	168	0.617	0.539
	Color × Illumination	4	168	0.844	0.498
Channel 38	Color	2	168	0.281	0.755
	Illumination	2	168	0.989	0.371
	Color × Illumination	4	168	3.195	0.016 *
Channel 39	Color	2	168	0.581	0.562
	Illumination	2	168	0.696	0.476
	Color × Illumination	4	168	1.093	0.358
Channel 40	Color	2	168	0.948	0.392
	Illumination	2	168	1.771	0.176
	Color × Illumination	4	168	0.500	0.723
Channel 41	Color	2	168	0.450	0.639
	Illumination	2	168	3.034	0.054
	Color × Illumination	4	168	1.139	0.339
Channel 42	Color	2	168	3.642	0.030 *
	Illumination	2	168	1.471	0.233
	Color × Illumination	4	168	2.102	0.087
Channel 43	Color	2	168	1.911	0.154
	Illumination	2	168	0.157	0.852
	Color × Illumination	4	168	0.621	0.646
Channel 44	Color	2	168	0.872	0.422
	Illumination	2	168	0.589	0.521
	Color × Illumination	4	168	1.388	0.248
Channel 45	Color	2	168	0.752	0.475
	Illumination	2	168	0.130	0.875
	Color × Illumination	4	168	0.449	0.770
Channel 46	Color	2	168	0.555	0.576
	Illumination	2	168	0.809	0.445
	Color × Illumination	4	168	0.827	0.508
Channel 47	Color	2	168	0.235	0.791
	Illumination	2	168	0.698	0.494
	Color × Illumination	4	168	0.179	0.945
Channel 48	Color	2	168	0.573	0.566
	Illumination	2	168	0.270	0.750
	Color × Illumination	4	168	0.985	0.414

* *p* < 0.05.

**Table 2:** ANOVA results for each Brodmann area.

Index	Effects	df_1_	df_2_	F	*p*
Left DLPFC	Color	2	168	0.042	0.959
	Illumination	2	168	1.104	0.327
	Color × Illumination	4	168	0.864	0.473
Right DLPFC	Color	2	168	0.472	0.625
	Illumination	2	168	1.044	0.351
	Color × Illumination	4	168	2.555	0.044 *
Left FPC	Color	2	168	0.623	0.539
	Illumination	2	168	4.161	0.018 *
	Color × Illumination	4	168	1.189	0.318
Right FPC	Color	2	168	2.926	0.059
	Illumination	2	168	1.514	0.224
	Color × Illumination	4	168	1.011	0.399
Left OFC	Color	2	168	0.430	0.652
	Illumination	2	168	0.801	0.437
	Color × Illumination	4	168	0.362	0.812
Right OFC	Color	2	168	0.077	0.926
	Illumination	2	168	0.298	0.737
	Color × Illumination	4	168	1.718	0.150
Left VLPFC	Color	2	168	0.963	0.386
	Illumination	2	168	0.522	0.580
	Color × Illumination	4	168	0.433	0.769
Right VLPFC	Color	2	168	1.656	0.197
	Illumination	2	168	0.469	0.622
	Color × Illumination	4	168	0.921	0.451

DLPFC, dorsolateral prefrontal cortex; FPC, frontopolar prefrontal cortex; OFC, orbitofrontal cortex; VLPFC, ventrolateral prefrontal cortex. * *p* < 0.05.

## Data Availability

The original contributions presented in this study are included in the article. Further inquiries can be directed to the corresponding authors.
